# Bathing

**Published:** 1856-12

**Authors:** 


### BATHING.

ONCE a week is often enough for a decent white man to wash himself all over, and whether in summer or winter, that ought to be done with soap, warm water, and a hog's-hair brush, in a room showing at least seventy degrees *Fahrenheit.* If a man is a pig in his nature, then no amount of washing will keep him clean, inside or out. Such an one needs a bath every time he turns round. He can do nothing neatly.

Baths should be taken early in the morning, for it is then that the system possesses the power of reaction in the highest degree. Any kind of bath is dangerous soon after a meal, or soon after fatiguing exercise. No man or woman should take a bath at the close of the day, unless by the advice of the family physician. Many a man, in attempting to cheat his doctor out of a fee, has cheated himself out of his life; aye, it is done every day.

The safest *mode* of a cold bath is a plunge into a river; the safest *time* is instantly after getting up. The necessary effort of swimming to shore compels a reaction, and the effect is delightful.

The best, safest, cheapest, and most universally accessible mode of keeping the surface of the body clean, besides the once-a-week washing, with soap, warm water, and hog's-hair brush, is as follows:

Soon as you get out of bed in the morning, wash your face, hands, neck, and breast; then, into the same basin of water, put both feet at once, for about a minute, rubbing them briskly all the time; then, with the towel, which has been dampened by wiping the face, feet, &c., wipe the whole body well, fast and hard, mouth shut, breast projecting. Let the whole thing be done within five minutes.

At night, when you go to bed, and whenever you get out of bed, during the night, or when you find yourself wakeful or restless, spend from two to five minutes in rubbing your whole body, with your hands, as far as you can reach, in every direction. This has a tendency to preserve that softness and mobility of skin which is essential to health, and which too frequent washings will always destroy.

That precautions are necessary, in connection with the bath-room, is impressively signified in the death of an American lady of refinement and position, lately, after taking a bath, soon after dinner; of Surgeon Hume, while alone, in a warm bath; and of an eminent New Yorker, under similar circumstances, all within a year.

